# 1-Butyl-4-hy­droxy-3-methyl­quinoline-2(1*H*)-one

**DOI:** 10.1107/S160053681004643X

**Published:** 2010-11-17

**Authors:** Zuzana Kozubková, Marek Nečas, Robert Vícha

**Affiliations:** a Department of Chemistry, Faculty of Technology, Tomas Bata University in Zlin, Nám. T. G. Masaryka 275, Zlín,762 72, Czech Republic; b Department of Chemistry, Faculty of Science, Masaryk University, Kamenice 5, Brno-Bohunice, 625 00, Czech Republic

## Abstract

In the crystal of the title compound, C_14_H_17_NO_2_, mol­ecules are arranged into chains along the *b* axis linked *via* O—H⋯O hydrogen bonds. While the benzene ring is essentially planar, with a maximum deviation from the best plane of 0.003 (1) Å, the pyridine ring is slightly V-shaped: the distance of the carbonyl C atom from the benzene best plane is 0.120 (1) Å. The hy­droxy group is inclined markedly towards the benzene ring reducing the C—C—O bond angle to 113.21 (10)°.

## Related literature

For the preparation, see: Stadlbauer & Kappe (1985 ▶). The title compound is a member of a group of substituted 4-hy­droxy­quinoline-2-ones used for preparation of new classes of heterocyclic systems, see: Klásek *et al.* (1998 ▶); Kafka *et al.* (2002 ▶).
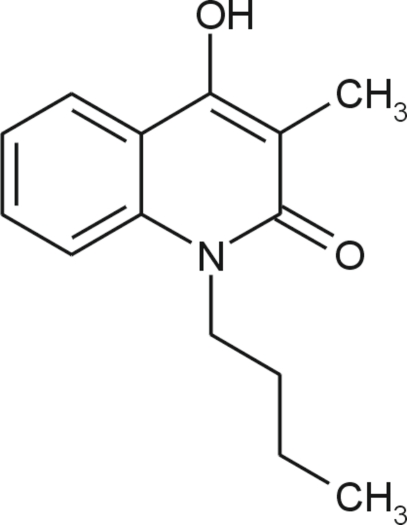


## Experimental

### 

#### Crystal data

C_14_H_17_NO_2_
*M* *_r_* = 231.29
Monoclinic, 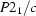

*a* = 11.8576 (7) Å
*b* = 10.7790 (6) Å
*c* = 9.8835 (7) Å
β = 110.749 (7)°
*V* = 1181.31 (13) Å^3^
*Z* = 4
Mo *K*α radiation
μ = 0.09 mm^−1^
*T* = 120 K
0.40 × 0.40 × 0.40 mm

#### Data collection

Oxford Diffraction Xcalibur diffractometer with a Sapphire2 detector
Absorption correction: multi-scan (*CrysAlis RED*; Oxford Diffraction, 2009 ▶) *T*_min_ = 0.978, *T*_max_ = 1.000
4533 measured reflections
2077 independent reflections
1625 reflections with *I* > 2σ(*I*)
*R*_int_ = 0.011

#### Refinement

*R*[*F* ^2^ > 2σ(*F* ^2^)] = 0.033
*wR*(*F* ^2^) = 0.086
*S* = 0.99
2077 reflections
157 parameters
H-atom parameters constrained
Δρ_max_ = 0.15 e Å^−3^
Δρ_min_ = −0.21 e Å^−3^

### 

Data collection: *CrysAlis CCD* (Oxford Diffraction, 2009 ▶); cell refinement: *CrysAlis RED* (Oxford Diffraction, 2009 ▶); data reduction: *CrysAlis RED*; program(s) used to solve structure: *SHELXS97* (Sheldrick, 2008 ▶); program(s) used to refine structure: *SHELXL97* (Sheldrick, 2008 ▶); molecular graphics: *ORTEP-3* (Farrugia, 1997 ▶) and *Mercury* (Macrae *et al.*, 2008 ▶); software used to prepare material for publication: *SHELXL97*.

## Supplementary Material

Crystal structure: contains datablocks global, I. DOI: 10.1107/S160053681004643X/zl2326sup1.cif

Structure factors: contains datablocks I. DOI: 10.1107/S160053681004643X/zl2326Isup2.hkl

Additional supplementary materials:  crystallographic information; 3D view; checkCIF report

## Figures and Tables

**Table 1:** Hydrogen-bond geometry (Å, °)

*D*—H⋯*A*	*D*—H	H⋯*A*	*D*⋯*A*	*D*—H⋯*A*
O2—H2*A*⋯O1^i^	0.84	1.86	2.6529 (14)	156

Symmetry code: (i) 
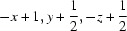
.

**Fig. 1.**
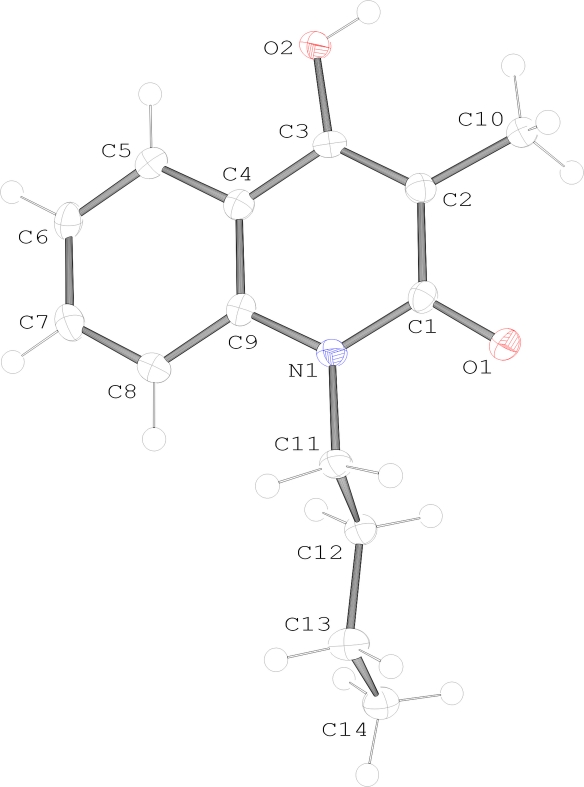
Anisotropic displacement view of the asymmetric unit with atoms represented as 50% probability ellipsoids.

**Fig. 2.**
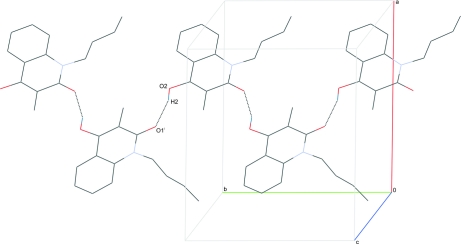
Part of the crystal structure showing chains linked via O—H···O hydrogen bonds along the b-axis. H-atoms have been omitted except for those participating in H-bonds. Symmetry code: (i) -x + 1, y + 1/2, -z + 1/2.

## supplementary crystallographic information

### Comment

Quinoline derivatives are well known and extensively studied especially for their wide occurance in nature and for their rich spectrum of biological activities. The title compound is a member of the group of substituted 4-hydroxyquinoline-2-ones used for preparation of new classes of heterocyclic systems (Klásek *et al.*, 1998; Kafka *et al.*, 2002).

The molecule of the title compound (Fig. 1) consists of fused benzene and pyridine rings. The benzene ring is essentially planar with a maximum deviation from the best plane of 0.0026 (12)Å for C6. The pyridine ring is slightly bent along the N1—C3 line with torsion angles C9—N1—C1—C2 and C4—C3—C2—C1 being 5.79 (17) and -6.38 (18)°, respectively. The geometry around C3 markedly differs from the ideal pattern for a *sp*^2^ carbon. All involved atoms C4–C2 and O1 lie in the plane of the phenyl ring (maximum deviation from the best plane is 0.0083 (12)Å for C3) but the valence angles C4—C3—O1 and C2—C3—O1 are 113.21 (10) and 125.91 (11)°. Molecules are linked *via* O2—H2···O1 H-bonds (Fig. 2, Table 1) into chains parallel to the *b*-axis.

### Experimental

Title compound was prepared according to a slightly modified procedure published by Stadlbauer & Kappe (1985). A mixture of *N*-butylaniline (16 cm^3^, 0.1 mol) and diethyl methylmalonate (17.2 cm^3^, 0.1 mol) was gradually heated in a Wood's metal bath at 413–553K for 6 h. The reaction was stopped when the amount of condensed ethanol reaches about 93% of the theoretical value. The hot mixture was poured on a metal plate and the crude product was quantitatively transferred into a 500 cm^3^ Erlenmeyer flask. After addition of 300 cm^3^ of 0.5 *M* NaOH and 50 cm^3^ of toluene the resulting mixture was stirred for 1 h. The suspension was extracted twice with 40 cm^3^ of toluene and the collected organic portions were treated with powdered activated carbon for 30 min at room temperature. The activated carbon was filtered off and approximately 300–400 cm^3^ of 5% HCl was added gradually into the filtrate. The precipitated crude product were filtered with suction and washed with water until neutral pH. Single crystals for X-ray analysis were grown by spontaneous evaporation from deuterochloroform at room temperature.

### Refinement

Hydrogen atoms were positioned geometrically and refined as riding using standard *SHELXL-97* facilities, with their U_iso_ set to either 1.2U_eq_ or 1.5U_eq_(methyl) of their parent atoms.

### Crystal data

**Table:**

C_14_H_17_NO_2_	*F*(000) = 496
*M**_r_* = 231.29	*D*_x_ = 1.300 Mg m^−^^3^
Monoclinic, *P*2_1_/*c*	Melting point: 471 K
Hall symbol: -P 2ybc	Mo *K*α radiation, λ = 0.71073 Å
*a* = 11.8576 (7) Å	Cell parameters from 2482 reflections
*b* = 10.7790 (6) Å	θ = 3.0–27.6°
*c* = 9.8835 (7) Å	µ = 0.09 mm^−^^1^
β = 110.749 (7)°	*T* = 120 K
*V* = 1181.31 (13) Å^3^	Block, yellow
*Z* = 4	0.40 × 0.40 × 0.40 mm

### Data collection

**Table:**

Oxford Diffraction Xcalibur diffractometer with a Sapphire2 detector	2077 independent reflections
Radiation source: Enhance (Mo) X-ray Source	1625 reflections with *I* > 2σ(*I*)
graphite	*R*_int_ = 0.011
Detector resolution: 8.4353 pixels mm^-1^	θ_max_ = 25.0°, θ_min_ = 3.0°
ω scans	*h* = −13→14
Absorption correction: multi-scan (*CrysAlis RED*; Oxford Diffraction, 2009)	*k* = −9→12
*T*_min_ = 0.978, *T*_max_ = 1.000	*l* = −11→11
4533 measured reflections	

### Refinement

**Table:**

Refinement on *F*^2^	Primary atom site location: structure-invariant direct methods
Least-squares matrix: full	Secondary atom site location: difference Fourier map
*R*[*F*^2^ > 2σ(*F*^2^)] = 0.033	Hydrogen site location: inferred from neighbouring sites
*wR*(*F*^2^) = 0.086	H-atom parameters constrained
*S* = 0.99	*w* = 1/[σ^2^(*F*_o_^2^) + (0.0533*P*)^2^] where *P* = (*F*_o_^2^ + 2*F*_c_^2^)/3
2077 reflections	(Δ/σ)_max_ = 0.001
157 parameters	Δρ_max_ = 0.15 e Å^−^^3^
0 restraints	Δρ_min_ = −0.21 e Å^−^^3^

### Special details

**Table:**

Geometry. All e.s.d.'s (except the e.s.d. in the dihedral angle between two l.s. planes) are estimated using the full covariance matrix. The cell e.s.d.'s are taken into account individually in the estimation of e.s.d.'s in distances, angles and torsion angles; correlations between e.s.d.'s in cell parameters are only used when they are defined by crystal symmetry. An approximate (isotropic) treatment of cell e.s.d.'s is used for estimating e.s.d.'s involving l.s. planes.
Refinement. Refinement of *F*^2^ against ALL reflections. The weighted *R*-factor *wR* and goodness of fit *S* are based on *F*^2^, conventional *R*-factors *R* are based on *F*, with *F* set to zero for negative *F*^2^. The threshold expression of *F*^2^ > 2σ(*F*^2^) is used only for calculating *R*-factors(gt) *etc*. and is not relevant to the choice of reflections for refinement. *R*-factors based on *F*^2^ are statistically about twice as large as those based on *F*, and *R*- factors based on ALL data will be even larger.

### Fractional atomic coordinates and isotropic or equivalent isotropic displacement parameters (Å^2^)

**Table:**

	*x*	*y*	*z*	*U*_iso_*/*U*_eq_	
O1	0.58070 (7)	0.83511 (7)	0.20280 (9)	0.0221 (2)	
O2	0.59308 (7)	1.27747 (7)	0.19762 (9)	0.0210 (2)	
H2A	0.5408	1.2746	0.2368	0.031*	
N1	0.68444 (8)	0.93464 (9)	0.08212 (10)	0.0165 (2)	
C1	0.61444 (10)	0.93675 (11)	0.16869 (12)	0.0168 (3)	
C2	0.58304 (10)	1.05481 (11)	0.21508 (12)	0.0164 (3)	
C3	0.62176 (10)	1.16166 (11)	0.17139 (12)	0.0161 (3)	
C4	0.70374 (10)	1.15797 (11)	0.09202 (12)	0.0166 (3)	
C5	0.75484 (10)	1.26662 (11)	0.06041 (13)	0.0194 (3)	
H5A	0.7342	1.3447	0.0898	0.023*	
C6	0.83446 (11)	1.26165 (12)	−0.01253 (13)	0.0220 (3)	
H6A	0.8688	1.3357	−0.0330	0.026*	
C7	0.86408 (10)	1.14718 (12)	−0.05593 (13)	0.0227 (3)	
H7A	0.9185	1.1437	−0.1070	0.027*	
C8	0.81590 (10)	1.03880 (11)	−0.02618 (13)	0.0195 (3)	
H8A	0.8376	0.9615	−0.0562	0.023*	
C9	0.73476 (10)	1.04230 (11)	0.04853 (12)	0.0164 (3)	
C10	0.50878 (10)	1.05049 (11)	0.31107 (13)	0.0206 (3)	
H10A	0.5068	1.1331	0.3516	0.031*	
H10B	0.5448	0.9913	0.3899	0.031*	
H10C	0.4264	1.0243	0.2542	0.031*	
C11	0.70685 (10)	0.81262 (11)	0.02898 (13)	0.0183 (3)	
H11A	0.7145	0.8233	−0.0669	0.022*	
H11B	0.6368	0.7580	0.0164	0.022*	
C12	0.82009 (10)	0.75039 (12)	0.13077 (13)	0.0198 (3)	
H12A	0.8072	0.7273	0.2212	0.024*	
H12B	0.8877	0.8103	0.1561	0.024*	
C13	0.85459 (11)	0.63463 (12)	0.06562 (14)	0.0251 (3)	
H13A	0.7849	0.5773	0.0332	0.030*	
H13B	0.8742	0.6585	−0.0203	0.030*	
C14	0.96221 (11)	0.56796 (11)	0.17331 (14)	0.0253 (3)	
H14A	0.9855	0.4982	0.1252	0.038*	
H14B	0.9405	0.5369	0.2540	0.038*	
H14C	1.0300	0.6258	0.2101	0.038*	

### Atomic displacement parameters (Å^2^)

**Table:**

	*U*^11^	*U*^22^	*U*^33^	*U*^12^	*U*^13^	*U*^23^
O1	0.0251 (5)	0.0147 (5)	0.0314 (5)	−0.0013 (4)	0.0161 (4)	0.0010 (4)
O2	0.0232 (5)	0.0150 (5)	0.0303 (5)	0.0007 (4)	0.0164 (4)	−0.0005 (4)
N1	0.0173 (5)	0.0139 (6)	0.0198 (5)	−0.0004 (4)	0.0085 (4)	−0.0015 (4)
C1	0.0153 (6)	0.0165 (7)	0.0178 (6)	−0.0014 (5)	0.0052 (5)	0.0013 (5)
C2	0.0139 (6)	0.0178 (7)	0.0170 (6)	0.0007 (5)	0.0047 (5)	0.0004 (5)
C3	0.0153 (6)	0.0140 (7)	0.0171 (6)	0.0015 (5)	0.0034 (5)	−0.0016 (5)
C4	0.0146 (6)	0.0176 (7)	0.0159 (6)	0.0005 (5)	0.0035 (5)	0.0011 (5)
C5	0.0208 (6)	0.0165 (7)	0.0201 (7)	0.0001 (5)	0.0061 (5)	0.0007 (5)
C6	0.0235 (6)	0.0205 (7)	0.0238 (7)	−0.0053 (5)	0.0107 (5)	0.0026 (6)
C7	0.0213 (6)	0.0279 (8)	0.0219 (7)	−0.0007 (6)	0.0113 (5)	0.0017 (6)
C8	0.0199 (6)	0.0201 (7)	0.0196 (7)	0.0016 (5)	0.0082 (5)	−0.0021 (5)
C9	0.0148 (6)	0.0177 (7)	0.0149 (6)	−0.0013 (5)	0.0032 (5)	0.0011 (5)
C10	0.0241 (6)	0.0156 (7)	0.0262 (7)	0.0004 (5)	0.0138 (5)	0.0002 (5)
C11	0.0207 (6)	0.0144 (7)	0.0216 (7)	−0.0020 (5)	0.0096 (5)	−0.0037 (5)
C12	0.0214 (6)	0.0176 (7)	0.0217 (6)	−0.0006 (5)	0.0093 (5)	−0.0011 (5)
C13	0.0265 (7)	0.0212 (7)	0.0262 (7)	0.0039 (6)	0.0074 (6)	−0.0021 (6)
C14	0.0282 (7)	0.0220 (7)	0.0271 (7)	0.0050 (6)	0.0115 (6)	0.0017 (6)

### Geometric parameters (Å, °)

**Table:**

O1—C1	1.2528 (13)	C8—C9	1.4056 (16)
O2—C3	1.3429 (13)	C8—H8A	0.9500
O2—H2A	0.8400	C10—H10A	0.9800
N1—C1	1.3873 (15)	C10—H10B	0.9800
N1—C9	1.3976 (14)	C10—H10C	0.9800
N1—C11	1.4749 (14)	C11—C12	1.5192 (16)
C1—C2	1.4454 (16)	C11—H11A	0.9900
C2—C3	1.3655 (16)	C11—H11B	0.9900
C2—C10	1.5064 (15)	C12—C13	1.5249 (16)
C3—C4	1.4499 (16)	C12—H12A	0.9900
C4—C5	1.4037 (16)	C12—H12B	0.9900
C4—C9	1.4095 (15)	C13—C14	1.5217 (16)
C5—C6	1.3771 (16)	C13—H13A	0.9900
C5—H5A	0.9500	C13—H13B	0.9900
C6—C7	1.3915 (17)	C14—H14A	0.9800
C6—H6A	0.9500	C14—H14B	0.9800
C7—C8	1.3772 (16)	C14—H14C	0.9800
C7—H7A	0.9500		
			
C3—O2—H2A	109.5	C2—C10—H10A	109.5
C1—N1—C9	122.14 (10)	C2—C10—H10B	109.5
C1—N1—C11	117.19 (9)	H10A—C10—H10B	109.5
C9—N1—C11	120.66 (10)	C2—C10—H10C	109.5
O1—C1—N1	118.00 (10)	H10A—C10—H10C	109.5
O1—C1—C2	122.81 (11)	H10B—C10—H10C	109.5
N1—C1—C2	119.19 (10)	N1—C11—C12	112.73 (9)
C3—C2—C1	119.28 (11)	N1—C11—H11A	109.0
C3—C2—C10	124.21 (11)	C12—C11—H11A	109.0
C1—C2—C10	116.51 (10)	N1—C11—H11B	109.0
O2—C3—C2	125.91 (10)	C12—C11—H11B	109.0
O2—C3—C4	113.21 (10)	H11A—C11—H11B	107.8
C2—C3—C4	120.86 (10)	C11—C12—C13	112.83 (10)
C5—C4—C9	119.36 (11)	C11—C12—H12A	109.0
C5—C4—C3	121.50 (11)	C13—C12—H12A	109.0
C9—C4—C3	119.12 (10)	C11—C12—H12B	109.0
C6—C5—C4	120.94 (12)	C13—C12—H12B	109.0
C6—C5—H5A	119.5	H12A—C12—H12B	107.8
C4—C5—H5A	119.5	C14—C13—C12	112.02 (10)
C5—C6—C7	119.38 (11)	C14—C13—H13A	109.2
C5—C6—H6A	120.3	C12—C13—H13A	109.2
C7—C6—H6A	120.3	C14—C13—H13B	109.2
C8—C7—C6	121.15 (11)	C12—C13—H13B	109.2
C8—C7—H7A	119.4	H13A—C13—H13B	107.9
C6—C7—H7A	119.4	C13—C14—H14A	109.5
C7—C8—C9	120.16 (11)	C13—C14—H14B	109.5
C7—C8—H8A	119.9	H14A—C14—H14B	109.5
C9—C8—H8A	119.9	C13—C14—H14C	109.5
N1—C9—C8	122.13 (11)	H14A—C14—H14C	109.5
N1—C9—C4	118.85 (10)	H14B—C14—H14C	109.5
C8—C9—C4	119.02 (11)		

### Hydrogen-bond geometry (Å, °)

**Table:**

*D*—H···*A*	*D*—H	H···*A*	*D*···*A*	*D*—H···*A*
O2—H2A···O1^i^	0.84	1.86	2.6529 (14)	156

Symmetry codes: (i) −*x*+1, *y*+1/2, −*z*+1/2.
